# Key populations are the future of the African HIV/AIDS pandemic

**DOI:** 10.1002/jia2.25750

**Published:** 2021-06-30

**Authors:** David Barr, Geoff P Garnett, Kenneth H Mayer, Michelle Morrison

**Affiliations:** ^1^ The Fremont Center New York NY USA; ^2^ Bill and Melinda Gates Foundation Seattle WA USA; ^3^ The Fenway Institute Beth Israel Deaconess Medical Center Harvard Medical School Boston MA USA; ^4^ Bill and Melinda Gates Foundation Seattle WA USA

**Keywords:** HIV, vulnerable populations, sub‐Saharan Africa, community‐based responses

1

The collective failure to invest sufficiently in comprehensive, rights‐based, people‐centred HIV responses has come at a terrible price. Implementing just the most politically palatable programmes will not turn the tide against COVID‐19 or end AIDS. To get the global response back on track will require putting people first and tackling the inequalities on which epidemics thrive.” Winnie Byanyima, Executive Director, UNAIDS [1]

Despite the progress made over the past 20 years towards reducing HIV infections, illness and deaths across much of sub‐Saharan Africa, the goal of “epidemic control” remains far from realized [1]. The global AIDS response was already off track before the COVID‐19 pandemic hit. The rapid spread of the coronavirus has created additional setbacks. Initial modelling suggested increases in mortality if HIV treatment was widely interrupted [2]. Thankfully, the worst assumptions were not realized. However, the coronavirus pandemic has substantially reduced HIV diagnoses and prevention uptake, which may increase HIV incidence over time [3]. These concerns continue to be salient given the inequitable access to COVID‐19 vaccines in Africa compared to the global North and reports of new COVID‐19 variants that may be less responsive to vaccines.

In reviewing the achievements of the global 2016 to 2021 HIV “Fast‐Track” strategy, UNAIDS has recognized that aggregate achievements in treatment, viral suppression and prevention mask poor results in some segments of the population, which undermine overall reductions in HIV incidence. As has been the case since the start of the epidemic, “key population” members – those at higher risk of HIV transmission including men who have sex with men, people who inject drugs, sex workers, migrants and the incarcerated – are being left behind. This special issue of the *Journal of the International AIDS Society* serves to document how the continued failure to adequately address the needs of these highly vulnerable, yet underserved populations will increasingly drive the course of the HIV pandemic in sub‐Saharan Africa over the coming years.

As with previous UNAIDS strategies, the newly proposed 2021 to 2026 Global AIDS Strategy emphasizes tackling inequalities, with equitable and equal access to HIV services, breaking down barriers to prevention and care and creating a strengthened, resilient and fully resourced response [4]. Similarly, UNAIDS released new HIV targets for 2025 [5]. However, the resources available for HIV programmes in low‐ and middle‐income countries (LMICs) fell short of the estimated needs of the previous Fast Track strategy. Annual shortfalls in resources required to fully fund the 2016 to 2021 strategy were cumulatively in excess of USD 20 billion [6]. At the end of 2019, USD 18.6 billion (constant 2016 dollars) was available for the AIDS response in low‐ and middle‐income countries, almost USD 1.3 billion less than in 2017. UNAIDS estimates that USD 26.2 billion (constant 2016 dollars) will be required for the AIDS response in 2020 [7]. The new strategy requires that investments increase to USD 30.9 billion by 2025, which will be challenging in the context of a global recession, a new global pandemic and sustainable development goals that are focused on health outcomes not specific to HIV.

The global rhetoric about ending the HIV pandemic as a public health threat by 2030 bears some examination. The goal is not HIV eradication, or local elimination, but a reduction in HIV incidence thereby reducing the burden of HIV infections. People living with HIV(PLHIV) will still require lifelong treatment and ongoing prevention interventions will still be required. HIV control cannot be achieved or sustained if the needs of the most vulnerable remain neglected. Over time, some populations will likely bear an even greater disproportionate burden of HIV. If HIV is treated less as an exception and integrated into universal health care, it is likely that funding of HIV services will be more aligned with funding of healthcare in general, while appropriate services for key populations could become even less available. Currently, just 2% of all HIV funding, and around 9% of resources allocated specifically for prevention, are spent on these groups [7]. The large majority of this funding comes from external donors. Unless domestic funding for key population HIV services is increased, epidemic control in many countries cannot be achieved.

Epidemic control has been defined as reaching a threshold where 73% of PLHIV are virally suppressed (the “third 90” in the 90% diagnosed, 90% in care, 90% effectively treated paradigm) which is expected to reduce incidence, or as reaching a threshold where HIV incidence is reduced below the rate of mortality of PLHIV [8]. These thresholds are meaningful in that the course of HIV epidemics may change if and when they are achieved, but higher levels of community viral suppression are still needed for faster and more sustained HIV control, and that “more” will require a greater concentration on the needs of vulnerable and key populations.

In sub‐Saharan Africa, HIV epidemic control cannot be achieved until and unless significantly greater focus and resources to meet the needs of those most vulnerable to HIV infection and illness – adolescents and young adults, and particularly key populations. Globally, it is currently estimated that 65% of new HIV infections occur within key population groups, and 17% of new HIV infections in East and Southern Africa (where the majority of all incident HIV infections are occurring) and 42% in West and Central Africa are occurring in key populations [9]. However, these are likely underestimates based on limited data, and the percentages are expected to continue to increase in the near future [10]. HIV infections among vulnerable populations do not occur in a vacuum but affect and will continue to affect the broader population.

Reductions in HIV incidence are likely to increase the importance of key population programmes for HIV control in sub‐Saharan Africa [11]. This special issue highlights interventions to better identify, support and retain these key populations in HIV prevention, treatment and care services with particular emphasis on community‐driven approaches.

Mworeko *et al*. [12] argue that communities of people living with and affected by HIV are the linchpin and entry point for greater success in reducing overall HIV risk. As epidemics evolve and change, refocusing and centering responses on communities becomes even more essential for shaping more effective HIV service delivery. This is not a new approach. The UNAIDS Fast Track strategy called for 30% of funding to go directly to communities for advocacy, mobilization and service delivery [13]. But since that strategy was launched, funding for community‐based advocacy and services has only decreased and never reached the recommended levels [14].

One foundational problem in understanding how to best shape HIV responses for key populations is the need to more accurately define the size of these populations, where they are located, what their risk behaviours are, who are their partners and what percentage of each country’s HIV infection and disease is attributable to these populations. The review by Jin *et al*. [15] provides an overview of the current epidemiology of HIV in key populations in sub‐Saharan Africa, illuminating the disproportionate disease burden and secular trends of HIV spread among them and underscoring the critical lack of data in many countries and contexts. Mishra *et al*. [16] focus on the measurement of the population attributable risk and how to account for the full impact of risk factors in understanding the evolution of African HIV epidemics. For example some key populations have very high HIV prevalence, but because of their small numbers, the impact of their disease burden on local epidemics will depend on their patterns of interactions with the general population. They offer a detailed analysis and summary of models to assess the attributable percentage of HIV infections throughout the region to these vulnerable populations over time. Such data are crucial to better allocate resources and target services where they are most needed.

In addition to the need for and value of improved demographic knowledge, there is also a need to better understand how individuals and communities define and organize themselves. Issues arise about the categories and labels that are used to explore the intersection between individuals and communities, and how people are viewed and treated. The label “key population” groups include people with a range of identities and characteristics. The label fails to acknowledge the complexity of individual identity while potentially increasing the risk of stigma and discrimination. And yet the label “key population” also provides opportunities to mobilize and express the opinions, needs and wants of people. The attitudes of society and organizations to those groups often determine whether the labels are useful or harmful. In addition, how we describe HIV epidemiology and the focus of interventions can promote or relegate the attention paid to key and vulnerable populations and can carry connotations of innocence and guilt. Such connotations are unhelpful if they undermine rights, prevent access to services and exacerbate the challenges of HIV. Thus, we need a careful balance between the clear description of epidemiological and cultural phenomena and the potential for misinterpretation or discrimination based on value judgements.

Placing people into demographic categories can oversimplify an individual’s identity. A sex worker is not only a sex worker, but can also be a mother, a father, a child, transgender, a migrant, a student and so on The lack of more sophisticated understanding of how multiple roles comprise a person’s identity results in lost opportunities to better engage people in health services and/or provide those services in ways that best meet their needs. In this regard, Makofane *et al*. [17] describe how subgroups of gay men and other men who have sex with men define themselves in different ways yet are able to find each other and develop support networks, which then turn into more formalized service delivery programmes.

Tapping into the knowledge and experiences of the most vulnerable themselves seems to be an obvious approach for policy making, but deeply ingrained hostility, stigma and discrimination against key populations often precludes such engagement from happening in a systematic way. Because of their unique experience and expertise in education, support, advocacy and mobilization, these communities need to be formally incorporated into HIV programming at all levels, with sufficient resources available. This is a longstanding challenge across sub‐Saharan Africa, as communities and key population networks face consistent funding and capacity challenges as well as resistance based on their perceived unprofessionalism. Several activities and solutions aimed at mitigating these challenges are presented. For example, Nibogora [18] describes programmes through which young gay men, many of whom are also sex workers, develop leadership skills in order to engage in national level programme and policy development.

Because stigma, discrimination and criminalization all heighten the risk of HIV infection and hinder the effective delivery of services, advocacy plays a central role in improving HIV health outcomes among key populations. Were *et al*. [19] and Caswell *et al*. [20] each discuss the importance of advocacy in meeting health, gender equity and human rights goals and present a variety approaches to such work, including community‐led monitoring of health services.

Keurohglian *et al*. [21] discuss the need for and methods to reduce stigmatization within healthcare settings through healthcare worker training, so that key population members will feel comfortable in accessing and persisting in care. Maruyama *et al*. [22] describe differentiated service delivery models utilizing peer‐based approaches to better engage key populations in treatment and prevention. Odhiambo [23] discusses effective methods to provide harm reduction services to people who use drugs. There is a clear role for governments to play in increased resource allocations for community‐based service delivery as well as scaling up interventions targeting key populations in general. Musoke *et al*. [24] describe how the government of Kenya has made strong and persistent efforts to scale up targeted services based on epidemiological data showing where and for whom HIV resources can have the greatest impact.

These examples point the way forward, but they are limited in scope and approach. Transformative change depends to a large extent on changes in policies and approaches among the most influential actors in HIV responses in the region. This is especially true in regard to financing. Interventions and programmes that more effectively identify, reach and support the most vulnerable can take time and money to design and implement. But they will save money over time if they result in declines in HIV infections, mortality and morbidity.

Potential approaches to unlock additional resources for such targeted work in sub‐Saharan Africa are discussed in this supplement. More strategic analysis and methodological emphasis in how and where resources from the US President’s Emergency Plan for AIDS Relief (PEPFAR) could direct more funding and support to key population‐specific prevention and support activities in many countries, as described by Jones, *et al*. [25]. Similarly, Long *et al*. [26] discuss how enhanced evidence‐based budget analysis in national and sub‐national finance ministries can show how and why more resource allocations should be directed towards the most vulnerable in order for overall funding to achieve the ultimate HIV response goals more efficiently and quickly.

The new UNAIDS 2021 to 2026 Global AIDS Strategy and 2025 targets prioritize addressing the needs of the most marginalized and putting affected communities in the forefront of the HIV response. As with previous targets and global strategies, these are laudable efforts. The failure to implement them will not only lead to further marginalization and stigmatization of key populations as they increasingly become the face of AIDS in Africa, but will undermine the goal of ending HIV as a public health threat. A true and well‐resourced commitment to meeting these targets can open the floodgates for the type of engagement and interventions needed, including those developed and led by communities, to better control HIV and save more lives.

## Competing interests

The authors have declared no conflict of interest.

## Authors’ contributions

The authors contributed equally to the content and development of this editorial.
